# Development of a Paper-based Hematocrit Test and a Lateral Flow Assay to Detect Critical Fibrinogen Concentrations Using a Bottom-Up Pyramid Workflow Approach

**DOI:** 10.1021/acsomega.3c10045

**Published:** 2024-02-05

**Authors:** Silvia Schobesberger, Helena Thumfart, Florian Selinger, Christoph J. Schlimp, Johannes Zipperle, Peter Ertl

**Affiliations:** † Faculty of Technical Chemistry, TU Wien, Getreidemarkt 9, 1060 Vienna, Austria; ‡ Ludwig-Boltzmann-Institute for Traumatology, The Research Center in Cooperation with AUVA, Donaueschingenstraße 13, 1200 Vienna, Austria; § Department of Anaesthesiology and Intensive Care, AUVA Trauma Center Linz, Garnisonstraße 7, 4010 Linz, Austria

## Abstract

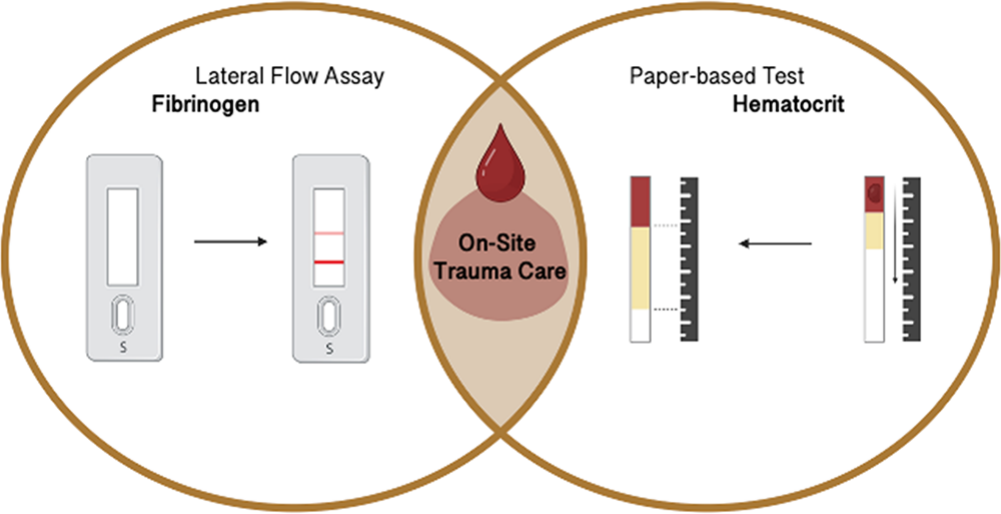

Fibrinogen is a coagulation factor in human blood and the first one to reach critical levels in major bleeding. Hypofibrinogenemia (a too low fibrinogen concentration in blood) poses great challenges to first responders, clinicians, and healthcare providers since it represents a risk factor for exsanguination and massive transfusion requirements. Thus, the rapid assessment of the fibrinogen concentration at the point of care has gained considerable importance in preventing and managing major blood loss. However, in whole blood measurements, hematocrit variations affect the amount (volume fraction) of plasma that passes the detection zone. In an attempt to accurately determine realistic critical levels of fibrinogen (<1.5 mg/mL) in patients needing immediate treatment and medical interventions, we have developed novel diagnostic systems capable of estimating hematocrit and critical fibrinogen concentrations. A lateral flow assay (LFA) for the detection of fibrinogen has been developed by establishing a workflow employing rapid characterization methods to streamline LFA development. The integration of two detection lines enables (i) the identification of fibrinogen (first line) present in the sample and (ii) the determination of the clinically critical fibrinogen concentrations below 1.5 mg/mL (second line). Furthermore, the paper-based separation of blood cells from plasma provides a semiquantitative estimate of the hematocrit by analyzing the fractions. Initial validation of the point-of-care (PoC) hematocrit test revealed good comparability to a standard laboratory method. The developed diagnostic systems have the ability to accelerate decision-making in cases with major bleeding.

## Introduction

Lateral flow assays (LFAs) represent a family of technological platforms for the rapid, inexpensive, and easy-to-use assessment of a variety of analytes on a membrane.^1^ As such, LFAs meet the criteria for point-of-care (PoC) testing summarized by the World Health Organization with the keywords: Affordable, Sensitive, Specific, User-friendly, Rapid and Robust, Equipment-free and Deliverable, in short, “ASSURED”.^2^ The origin of lateral flow tests goes back to the 1950s when paper-based dipstick tests for glucose measurements in urine were developed, and in parallel, latex agglutination assays^3^ and radioimmunoassay,^4^ precursors of the detection mechanism, emerged. The first LFA, similar to those available today, was developed in the 1980s to detect the presence of human chorionic gonadotropin (hCG) in urine to confirm pregnancy.^5^ Since then, lateral flow tests have become successful analytical devices for PoC testing in various settings,^6^ ranging from detecting disease biomarkers,^7,8^ pathogenic bacteria,^9^ viruses,^10^ and mycotoxins^11^ to chemical contaminants like veterinary drug residues or pesticides.^12^ Since the SARS-CoV-2 outbreak in 2019, LFAs have become one of the most abundant diagnostic tools for identification and monitoring of virus spread, needed to implement quarantine measures, despite their analytical drawbacks.^13^ For instance, LFAs mainly provide qualitative or semiquantitative results with sensitivities and specificities significantly lower than standard laboratory tests (such as PCR and ELISA).^1,14,15^ To overcome these analytical challenges, a range of strategies for improvement have been extensively investigated in the last years.^16^ A number of reviews have exclusively focused on the increase of assay sensitivity and mention the (i) improvements of pad geometries,^17^ (ii) controlling the flow speed,^1,18,19^ and (iii) engineering novel detection labels,^16^ among others. In addition to the variety of reviews, more than 2000 articles about LFAs were published in the past decade.^6^ Interestingly, only a handful of articles discuss methods that enable the rapid development of a LFA or the optimization of established LFAs.^15,20,21^ This aspect, however, is of increasing importance in the field since LFA development using a streamlined workflow reduces time-consuming research and development phases and additionally leads to improved assay performance and diagnostic outcomes. One example where accurate assay performance in PoC diagnostic applications is particularly important involves traumatic injuries where medical interventions such as fibrinogen-supplementation are known to decrease the risk of major hemorrhage,^22^ a leading cause of mortality of trauma patients.^23,24^ Fibrinogen plays an important role in stopping bleeding because this protein enhances initial platelet based blood clots through the formation of fibrin.^25^ In the routine clinical laboratory-based practice, the Clauss method, relying on the clot formation time of plasma, is used to measure fibrinogen concentrations.^26^ In order to measure the functional fibrinogen, viscoelastic tests, measuring mechanical property changes during clot formation,^27^ are employed. However, to enable clinical decision-making at the point of need, accurate, rapid, and reliable diagnostic approaches to determine the (functional) fibrinogen concentration are required.^22,28,29^ So far, developed PoC devices for the detection of fibrinogen in whole blood/plasma mainly rely on physical property changes related to coagulation. By adding thrombin, fibrin formation is induced, and consequently, the flow behavior of the sample or eluent within a membrane changes.^22,29−31^ Another more sophisticated approach employs screen-printed paper electrodes to separate blood cells by dielectrophoretic force and, after that, measures the fibrinogen concentration based on a resistance change after thrombin application leads to fibrin formation.^28^ To our knowledge, no lateral flow immunoassay for the detection of critical fibrinogen concentrations has been reported, which may be associated with difficulties in fine-tuning the assay for detecting a critical concentration. Compared to paper-based assays that rely on fibrin formation,^29−31^ the detection of the fibrinogen concentration using a LFA has the advantage of encountering misleading results due to the intake of anticoagulants. As an example, heparin, an anticoagulant drug with an inhibitory effect on thrombin, was shown to affect the measured fibrinogen concentration in a paper-based assay,^31^ but the effect is reduced when the blood sample is diluted.^30^ However, when using a LFA for the fibrinogen detection, the LFA’s inability to account for hematocrit variations in the blood sample needs to be addressed. Hematocrit, the percentage of blood cells in comparison to the plasmatic fraction, will naturally affect the availability of the surpassing plasma analyte at a fixed-volume ratio. Consequently, a higher hematocrit means a lower volume of plasma passing the detection zone, which ultimately affects the result. For a coagulation assay, Li et al. reported that the result is affected by the blood’s hematocrit and therefore developed a lateral flow device to determine the hematocrit.^32,33^ In addition, paper-based devices have been reported, showing a linear correlation between a blood’s hematocrit and travel distance in a wax-printed channel.^34,35^ Other developed PoC methods for hematocrit measurement include portable smartphone-based analytical platforms^36,37^ or a portable centrifugal device.^38^ Thus, when fibrinogen is measured at a fixed blood volume, the sample’s hematocrit should be taken into account. With regard to clinical decision-making, patients who are incorrectly diagnosed with hypofibrinogenemia may undergo unnecessary medical interventions, while trauma patients with incorrect physiological levels of fibrinogen may not undergo fibrinogen supplementation, thus increasing the risk of hemorrhage.

**Figure 1:**
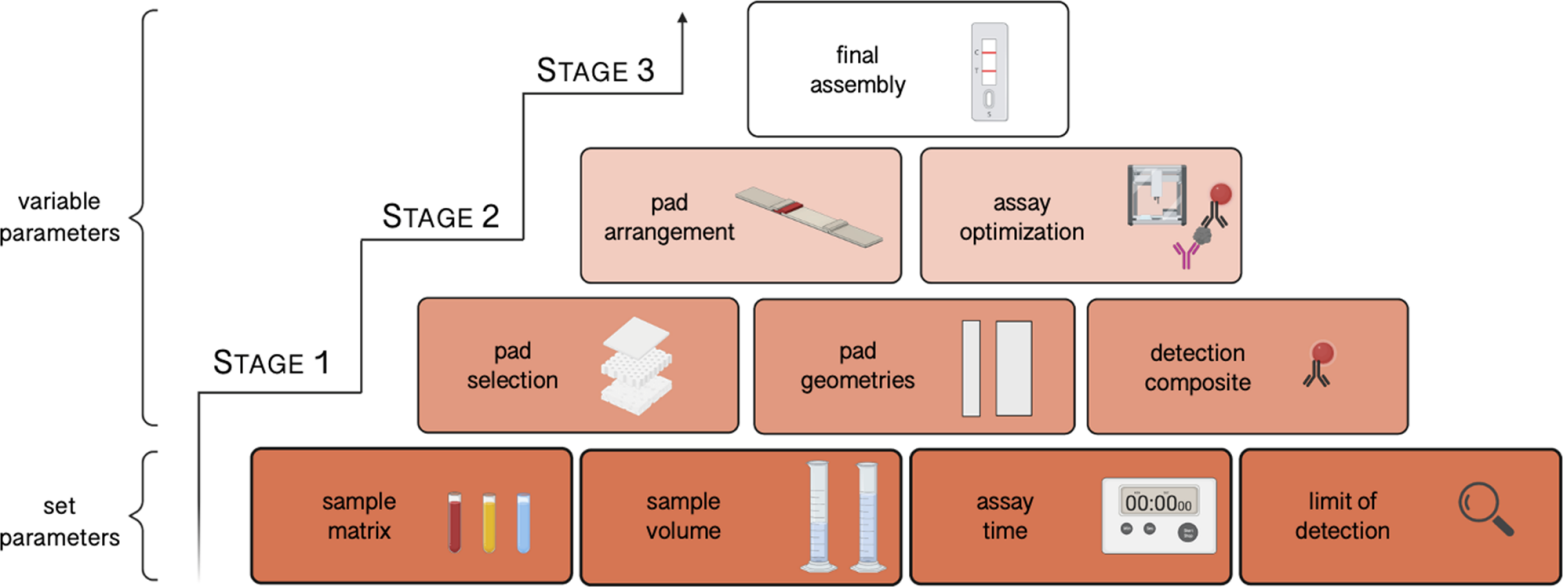
Workflow for the development of a fibrinogen LFA. Depending on the defined parameters, the pad, geometries, and detection composite are selected and tested (stage 1). Next, pads are combined and concentrations optimized (stage 2). In the end, the final test strip is assembled (stage 3).

In order to complement the existing fibrinogen PoC testing systems, we have developed a novel LFA that simultaneously (i) detects the presence of fibrinogen in whole blood samples and (ii) identifies whether fibrinogen has reached a critically low level. This has been accomplished by introducing an improved workflow that can be applied as a general guide for LFA development (see also Figure 1), focusing on material properties and its characterization but not in detail on immunoassay development. In addition, we developed a paper-based method to estimate the hematocrit. Our platform combination of a PoC hematocrit test and fibrinogen LFA would allow healthcare professionals to take the appropriate measures and interventions in an emergency setting more quickly.

## Results and Discussion

### Defining the Requirements of the Test to Be Developed

In general, the application of the workflow is streamlined for the development of a LFA detecting fibrinogen; however, a more general description of the proposed workflow can be found in the Supporting Information. At the beginning of the development, the workflow proposes to define precisely the requirements of the test to be developed. Sample matrix composition, sample volume, limit of detection, and assay time need to be determined for the analyte of interest and targeted application. In this case, since the target analyte fibrinogen is circulating at high concentrations in the bloodstream, blood was chosen as the sample matrix. To perform the test rapidly (ideally) with blood from a finger prick, a sample volume of 20 μL and a time-to-result of 10 min was selected. The detection threshold was set at 1.5 mg/mL because fibrinogen concentrations below this level are known to be critical for the patient and indicate the necessity of its supplementation.^39^ In addition, the most appropriate assay format, such as sandwich or competitive, needs to be selected. A sandwich assay is commonly used for big molecules like antibodies or proteins, while a competitive assay is used for small molecules or peptides.^37^ In our case, a sandwich assay was selected to detect fibrinogen with a molecular weight of around 340 kDa.

### Stage 1: Pad Characterization and Development of the Detection Composite

#### Detection Pad Selection and Characterization of Flow Modulation

**Figure 2:**
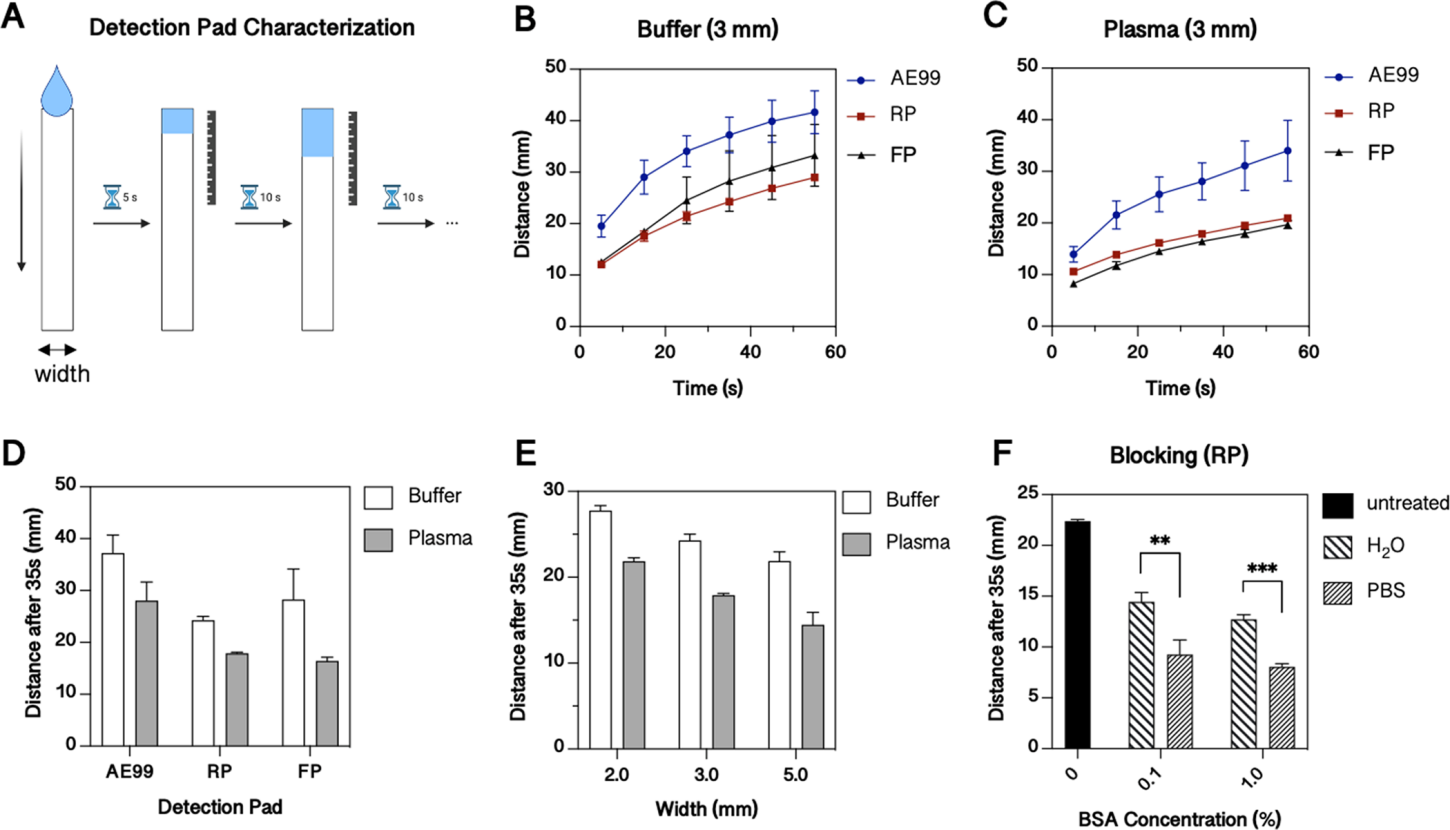
Characterization of the detection pads’ properties with buffer (Hank’s Balanced Salt Solution, HBSS) and plasma. (A) Schematic of the used method for characterization. Flow profile of different detection pads (AE99, RP, and FP) after applying (B) buffer or (C) plasma. (D) Comparison of travel distance after 35 s (width = 3 mm). (E) Effect of the membrane width (detection pad: RP). (F) Effect of blocking with bovine serum albumin (BSA) prepared in distilled water (H_2_O) or phosphate buffered saline (PBS) (detection pad: RP; buffer). Statistical significance by Welch *t-*test *p < 0.033, **p < 0.002, ***p < 0.001 (*n* = 3).

The detection pad, usually a nitrocellulose membrane, efficiently binds proteins.^1^ In total, three different nitrocellulose membranes, two with a plastic backing (RP and FP) and one unbacked (AE99) (see Table 1), were characterized with buffer and plasma samples to study the effect of (i) membrane composition, (ii) geometry, and (iii) blocking solutions on the flow profile. All experiments used aliquots of 10 μL sample to simulate the approximate plasma volume generated after red blood cell separation in 20 μL whole blood samples. At this point, it is important to note that in the testing setup, hydrostatic sample loading over a period of 5 s was used instead of capillary forces present in the final LFA assembly (Figure 2A). The flow study results are shown in Figure 2B,C, where the distances of the flow front were determined optically every 10 s. In general, the nonlinear flow velocity decreased over time but was elevated for AE99. A direct flow comparison between buffer and plasma samples further revealed that the plasma flow velocity was generally slower independent from the employed membranes (Figure 2D), which can be attributed to the higher viscosity of plasma.^40^ Overall, the lowest standard deviation of 17.9 ± 0.19 mm was found for the RP membrane (see also Figure 2D) compared to 28.1 ± 3.56 mm for AE99 and 16.4 ± 0.68 mm for FP. Consequently, RP was selected as the detection pad for all remaining experiments. Interestingly, the plasma travel distance of the AE99 membrane is 1.6- and 1.7-fold increased compared to those of RP and FP, respectively. This aspect may be attributed to the fact that AE99 is an unbacked membrane immobilized on a hydrophilic adhesive, which affected the apparent flow rate. Additional factors, such as geometry and blocking, were investigated to study the impact of reduced widths and the blocking agent bovine serum albumin (BSA) to gain a deeper understanding of flow behavior modulation. Results of flow behavior experiments using the three nitrocellulose membranes cut to 2, 3, and 5 mm width are shown in Figure 2E. A clear linear correlation between membrane width and flow rate was found, indicating that in the presence of small sample volumes, such as in finger prick applications, narrower membrane dimensions can help to increase the total length of the assembled membranes within the LFA device. Since narrower test strips also facilitate a faster flow profile, the interaction time between the analyte and the immunoconjugate can be reduced, thus offering the ability to adjust linear detection ranges and saturation levels. Another parameter that may influence a membrane’s flow behavior is the addition of blocking agents such as BSA. Since BSA is commonly used in membrane pretreatment applications to avoid unspecific adsorption, it is important to understand its effect on overall fluid flow. The RP nitrocellulose membrane was soaked in solutions with increasing BSA concentrations of 0–0.1–1% and the travel distances after 35 s were recorded. The result in Figure 2F reveals that the blocking decreases the flow speed by 35 and 43% for 0.1 and 1% BSA in water, respectively. In contrast, BSA dissolved in PBS reduced the flow velocity even more, up to 59 and 64% for 0.1 and 1% BSA, respectively, thus indicating that as an example the salt content exhibits an additional blocking effect. These results point to the ability to carefully adjust flow rates by employing coatings, increasing salt concentrations, and reducing membrane geometries.

**Table 1:** Overview of the Tested Pads and Their Characteristics Stated by the Vendor

	pad	material	thickness	flow rate	additional information
sample pad	GF	asymmetric polysulfone	330 μm	n.a.	untreated
					void volume: 1×
	GX	asymmetric polysulfone	330 μm	n.a.	post-treatment
					void volume: 1–1.5×
	GR	asymmetric polysulfone	330 μm	n.a.	post-treatment
					void volume: 2–2.5×
	LF1	bound glass fiber	247 μm (at 53 kPa)	35.6 s/4 cm	untreated
conjugate pad	ST14	bound glass fiber	355 μm (at 53 kPa)	23.1 s/4 cm	water absorption: 50.9 mg/cm^2^
	ST17	bound glass fiber	370 μm (at 53 kPa)	34.5 s/4 cm	water absorption: 44.9 mg/cm^2^
	Fusion 5	proprietary single layer matrix membrane	370 μm (at 53 kPa)	38 s/4 cm	water absorption: 40 mg/cm^2^
detection pad	FP	nitrocellulose membrane	100 μm (w.o. backing)	140–200 s/4 cm	plastic backing (polyester)
	RP	nitrocellulose membrane	100 μm (w.o. backing)	90–150 s/4 cm	plastic backing (polyester)
	AE99	nitrocellulose membrane	120 μm	120–160 s/4 cm	unbacked

#### Optimization of Gold Nanoparticle Conjugation and Conjugate Pad Release

**Figure 3:**
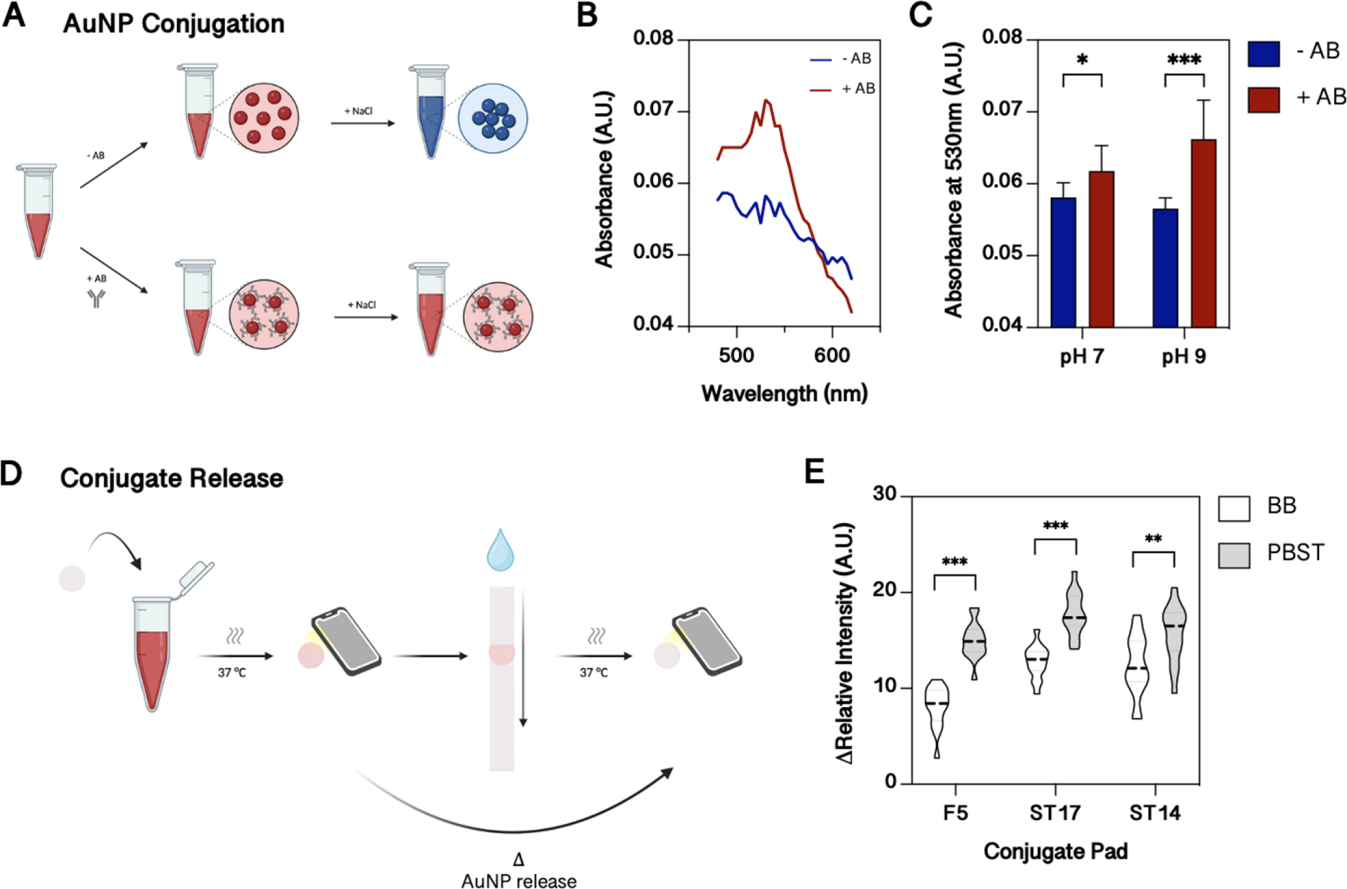
(A) Schematic of the method used to test the effectiveness of the conjugation of gold nanoparticles (AuNPs) with antibodies. If AuNPs are modified with antibodies (AB), the aggregation of AuNPs in the presence of NaCl is prevented. (B) Spectrum scan of bare (blue) and antibody-modified (red) AuNPs in the presence of NaCl. (C) Effect of the pH of the conjugation buffer on the conjugation of antibodies with AuNPs. (D) Schematic of the used method to study release properties of conjugate pads. The conjugate pad is saturated with AuNPs and an image is taken after the pad is dried. Next, stored conjugates are released and imaged again after the pad is dried. The intensity difference describes the AuNP release. (E) Conjugate release of different conjugate pads (F5: Fusion 5, ST17, ST14) and conjugate release buffer (BB: 5 mM borate buffer pH 9 with 10% sucrose, PBST: phosphate buffered saline with 0.05% TWEEN® 20 and 10% sucrose). (C,E) Statistical significance by Welch *t*-test *p < 0.033, **p < 0.002, ***p < 0.001 (*n* = 9 (C), *n* = 15 (E) from 3 independent experiments).

AuNPs are still widely used in LFAs as universal optical readout labels, but they need to be first modified with detection antibodies and then characterized. In Figure 3A an easy-to-use method with rapid quality control is proposed to evaluate the employed conjugation chemistry using commercially available AuNPs. It is well-known that AuNPs aggregate in the presence of a high salt concentration, resulting in a color shift from red to blue.^41^ However, if antibodies or proteins are conjugated to the AuNP, then aggregation is not possible anymore. To test this one-step analytical approach, absorption spectra of bare (blue trace) and fibrinogen antibody decorated (red trace) 40 nm AuNPs were recorded in the presence of sodium chloride. Results in Figure 3B show an absorption maximum of 530 nm only in the presence of the non-aggregated nanoparticles, thus verifying this spectroscopic analysis method. Since the conjugation efficiency further improves when the pH is close or slightly above the isoelectric point of the conjugation protein,^42^ different pH values were tested in subsequent experiments. The result of the comparative study is shown in Figure 3C and revealed a significant difference between pH 7.4 (PBS) and 9 (borate buffer), indicating the generation of more stable conjugates at pH 9.

Another important aspect in the development of a LFA is the release of the selected AuNP conjugates from the pad as soon as the sample enters. Therefore, two glass fiber pads (ST14 and ST17) and a proprietary material Fusion 5 (F5) were initially tested using commercially available AuNP streptavidin conjugates as low-cost model conjugates. For that, the conjugates were dried within the conjugate pad, and the intensity of the conjugate pad was analyzed before and after the release (Figure 3D). In addition, the effect of borate buffer and PBS with TWEEN® 20 (PBST), both supplemented with 10% sucrose to increase antibody stability and foster efficient conjugate release,^43^ were investigated in more detail. As shown in Figure 3E, the highest intensity difference (before and after the release) was observed for ST17, while PBST released the conjugates significantly better than the borate buffer in all cases (1.4-fold higher release for ST17). Therefore, we decided to continue with ST17 for the final LFA setup.

#### Sample Pad Characterization and Hematocrit Test Development

**Figure 4:**
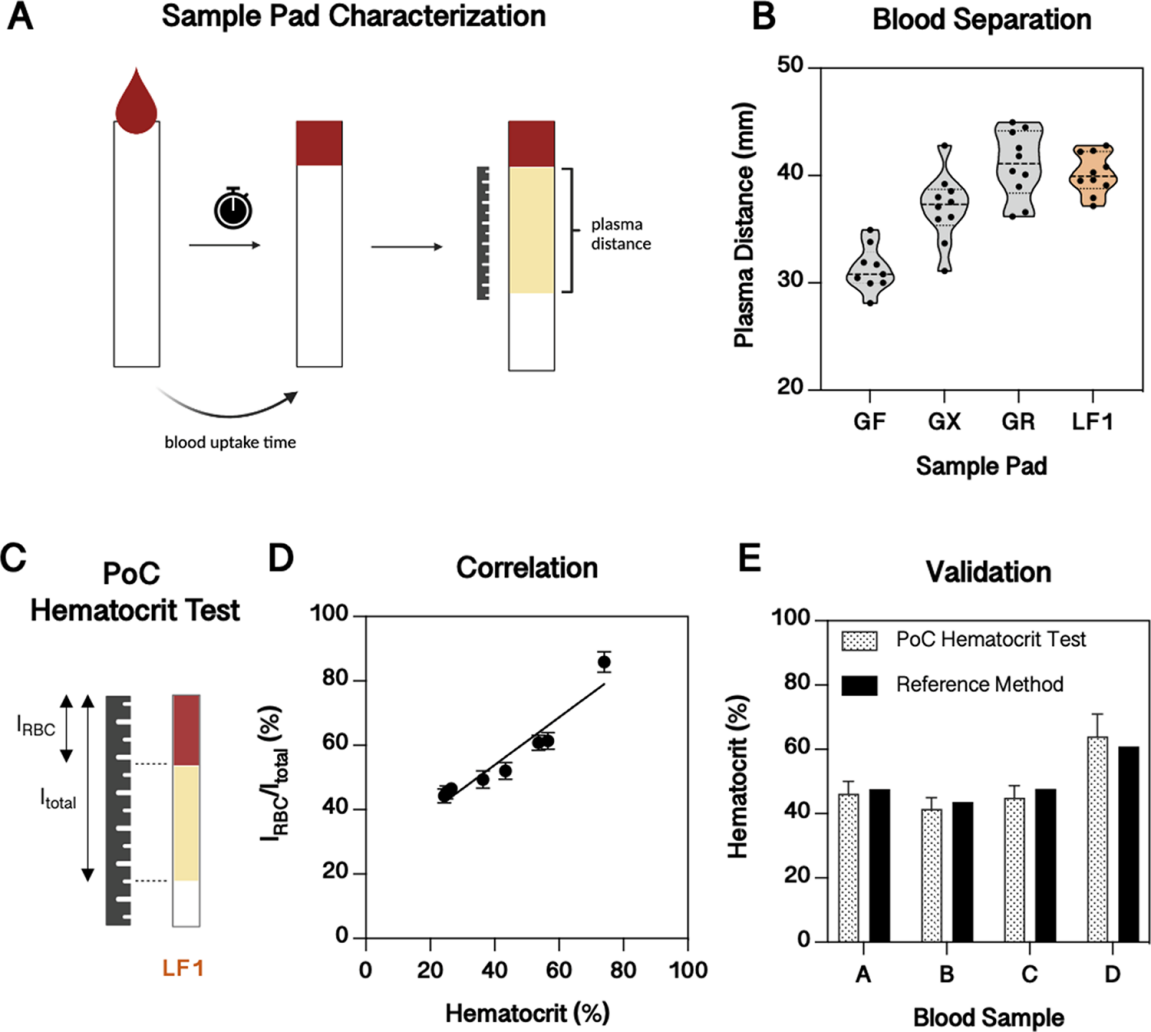
Sample pad characterization and point-of-care (PoC) hematocrit test development. (A) Schematic of the method used to characterize different sample pads (GF, GX, GR, LF1). (B) Plasma distance of different sample pads (width = 3 mm) after application of 20 μL of blood (*n* = 10). LF1 is colored orange since it was used for the PoC hematocrit test. (C) Schematic of the working principle of the PoC hematocrit test (*l*_RBC_ = travel length of red blood cells; *l*_total_ = travel length of red blood cells and plasma). (D) Correlation between PoC hematocrit test and hematocrit determined by hematology analyzer of samples prepared with different hematocrits. (E) Hematocrit of unknown blood samples determined with the developed PoC hematocrit test and a hematology analyzer (reference method).

Sample pad material characterization was conducted to determine the pad’s ability to retain red blood cells, which is crucial for efficient fibrinogen labeling and detection. In a comparative study, a glass fiber separator (LF1) and three asymmetric polysulfone membranes (GX, GF, and GR), varying in the void volume and chemical treatment of the membrane, were analyzed. The two membrane types differ in their separation mechanism since LF1 separates horizontally, whereas GX, GF, and GR separate vertically. Figure 4A shows a schematic drawing of the method used. A blood volume of 20 μL was added, and the blood uptake time determined (Figure S1) as well as the plasma extraction was studied by measuring the running distance of the plasma.

Besides identifying the membrane with the best separation efficiency, the determined running distance of the plasma is used to adjust the pad sizes accordingly. Comparing the asymmetric polysulfone membranes, GR generated the most plasma and was comparable to LF1 (Figure 4B). In addition, the area covered with red blood cells was 44 ± 2.6% for LF1 and 46 ± 2.5% for GR, indicating a very efficient separation since the blood sample’s hematocrit was 41%. We decided to use GR for the final LFA since it separates blood efficiently and soaks up the blood faster than LF1.

The plasma volume passing the detection zone in the fibrinogen LFA depends on the blood sample’s hematocrit. To develop a test method for hematocrit determination, we utilized the blood separation properties of the sample pad. Although GR and LF1 were comparable in separation efficiency, we used the LF1 for the hematocrit determination since it separates horizontally, and thus red blood cells and plasma are clearly visibly distinguishable, whereas the GR needs to be flipped to see the distance of the plasma. To determine the hematocrit, a similar analysis method is used as applied in glass capillary hematocrit measurements, where the capillary is filled with blood, and the ratio of red blood cells to plasma is determined after centrifugation. In our PoC hematocrit test, blood is added to the sample pad, separated by the blood separation membrane, and the ratio of red blood cells (*l*_RBC_) and total travel distance (*l*_total_) is determined, as depicted in Figure 4C. This approach is further characterized by measuring samples with increasing hematocrit and benchmarked against hematocrit values obtained by a hematology analyzer. As shown in Figure 4D, the obtained linear correlation allows us to determine the hematocrit with the developed PoC approach. For validation, we measured the hematocrit of four unknown blood samples and compared the results with a reference laboratory-based method, a hematology analyzer, showing good comparability (Figure 4E) for our PoC hematocrit test.

### Stage 2: Pad Arrangement and Assay Optimization

#### Development of a Lateral Flow Assay for the Detection of Fibrinogen

**Figure 5:**
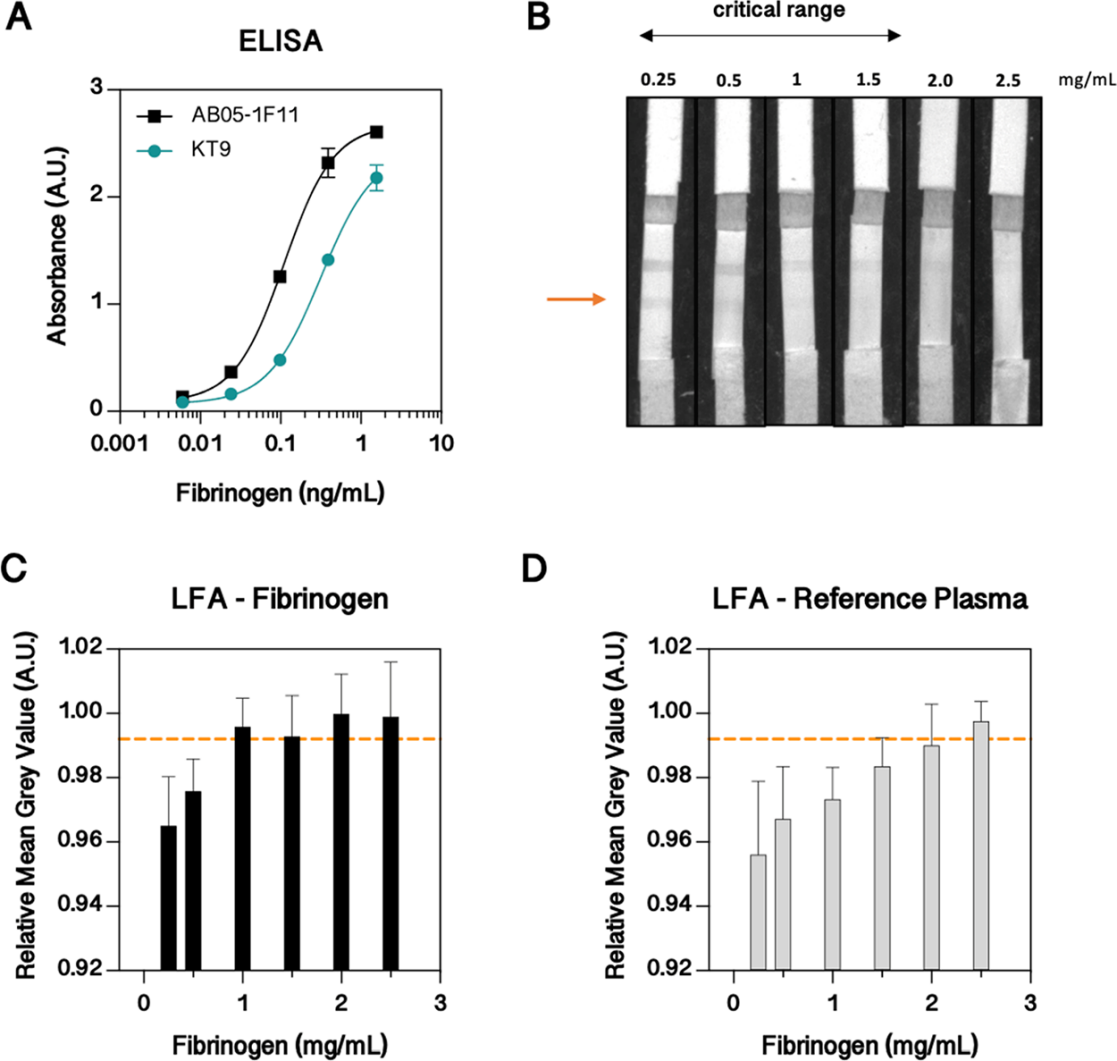
Fibrinogen lateral flow assay (LFA) development. (A) ELISA with two different capture antibodies (clone KT9 and AB05-1F11). (B) Images of LFA tested with different fibrinogen concentrations. The second line (indicated with an orange arrow) is used to determine the signal intensity. If the line appears, then the fibrinogen concentration is below the threshold of 1.5 mg/mL. (C) LFA tested with different fibrinogen concentrations. The orange line indicates a threshold of 1.5 mg/mL (*n* ≥ 6 from 3 independent experiments). (D) LFA tested with different dilutions of reference plasma. The orange line indicates a threshold of 1.5 mg/mL (*n* = 6 from 2 independent experiments).

Since a sandwich assay is employed to detect a big molecule, such as fibrinogen, ELISA tests were initially performed to identify a suitable antibody pair. In a comparative analysis, two different capture antibodies in combination with a biotinylated detection antibody were tested. Results in Figure 5A show that the antibody AB05-1F11 exhibited higher absorbance values when the same concentrations were tested, meaning that more sandwich complexes were formed, and therefore this capture antibody was used further. When transferring this sandwich assay to the membranes, we observed that the labeled AuNPs were immediately captured in the detection pad after release. We assumed that fibrinogen binds to the nitrocellulose membrane since it did not occur when fibrinogen was absent. Consequently, we blocked the membrane to avoid unspecific binding, however, the resulting flow rate decreased, and the high abundance of fibrinogen clogged the membrane completely at high concentrations. Thus, the sample was diluted 3-fold prior to sample application to lower the protein concentration and thereby ensure proper fluid flow. Interestingly, higher fibrinogen concentrations resulted in lower band intensities, and vice versa, lower fibrinogen concentrations resulted in more intense lines (Figure 5B). However, if the sample and conjugate were added separately, the line also appeared when high fibrinogen concentrations were added, indicating that due to the high fibrinogen concentration, released AuNP conjugates, as well as the immobilized capture antibody, are saturated with fibrinogen (hook effect). This observation suggests that fibrinogen without conjugate travels faster than with the conjugate and thus saturates the capture antibody, which is further supported by the fact that the intensity of the line depends on the line’s position. The lines close to the conjugate pad exhibited higher intensities than those further downstream. Therefore, two identical lines were integrated into the LFA to determine both the presence of fibrinogen and its concentration. The line close to the conjugate pad indicated the presence of fibrinogen, and the second line was used to determine the concentration (Figure 5B). Results in Figure 5C show the LFA with increasing fibrinogen concentrations of 0.25–2.5 mg/mL. Fibrinogen concentrations below 1 mg/mL were under the set threshold of 1.5 mg/mL, whereas 1 mg/mL was still in the threshold’s range. Since increased matrix complexity results in signal intensity changes, the LFA was validated with reference plasma, with a known fibrinogen concentration of 2.5 mg/mL. Critically relevant concentrations such as 0.25, 0.5, and 1 mg/mL fibrinogen were below the threshold, demonstrating the applicability of the developed LFA. Interestingly, especially at fibrinogen concentrations above the critical range, the contrast between background and line decreases. Thus, for image analysis, the line intensity was related to the background, underlining the importance of an automated image analysis integrated into a PoC reader (e.g., smartphone).

### Stage 3: Final Assembly

This work focused on the development of a LFA for the detection of fibrinogen and a PoC hematocrit test. The integration into a single device describes one of the next steps, which needs to be adapted to the requirements of a PoC reader since both diagnostic approaches rely on image analysis. However, the development of the PoC readout unit, consisting of a reader or smartphone application and an adapted casing, was not within the scope of this study. For feasibility, a 3D-printed casing with an integrated scale (Figure S5) for the PoC hematocrit test was already developed.

## Conclusions

In this work, we present the establishment of a LFA-based platform for the detection of critical levels of fibrinogen and propose a combination with a PoC hematocrit test. We applied a novel pyramid-shaped workflow to develop this rapid and accurate LFA for whole blood fibrinogen detection and determination of critical concentrations. We emphasize the importance of a workflow for structured development, but depending on the study’s scope, blocs as well as the volume of work for each stage may vary. Our study focused on stage 1 by characterizing the different pads to get an overview of the available toolbox to fine-tune the assay. A major highlight of this study is that sample pads were not only characterized regarding the highest blood separation efficiency but the pads’ properties were also used to develop a PoC hematocrit test. This paper-based method allowed rapid determination of the hematocrit of a blood sample, and initial results showed a good correlation with a standard laboratory method. A LFA for the detection of fibrinogen was developed that identified clinically critical fibrinogen concentrations below 1.5 mg/mL. This threshold is important since the treatment strategy needs to be adapted if fibrinogen concentrations are lower. Combining the fibrinogen LFA and the PoC hematocrit test, critical fibrinogen concentrations can be determined directly next to the patient at the point of need, enabling faster adaptation of the treatment plan. To further improve the applicability of the diagnostic system, we want to develop a readout unit for automated image analysis. A prospective clinical investigation will be undertaken to validate the robustness of the developed methods.

## Experimental Section

### Materials

Phosphate buffered saline (PBS), Hank’s Balanced Salt Solution (HBSS), 40 nm gold nanoparticles (AuNPs), borate buffer (0.5 M, pH 9), bovine serum albumin (BSA), TWEEN® 20, 3,3′,5,5′-tetramethylbenzidine, and sucrose were purchased from Merck (Rahway, NJ, US); 40 nm AuNP modified with streptavidin was obtained from Abcam (Cambridge, GB). Horseradish peroxidase (HRP) conjugated streptavidin was purchased from Thermo Fisher Scientific (Hampton, NH, US). Cytiva (Marlborough, MA, US) provided Whatman blood separator LF1, Whatman nitrocellulose membranes Immunopore FP, Immunopore RP, AE99, and the conjugate pads Whatman Standard 14 (ST14), Standard 17 (ST17), and Fusion 5 (F5). In addition, Pall Corporation (NY, US) provided the Vivid plasma separation membranes GF, GX, and GR. The adhesive tape ARflow 90469 was procured from Adhesive Research (Glen Rock, PA, US). The anti-human fibrinogen monoclonal antibodies were purchased from Thermo Fisher Scientific (Hampton, NH, US), Bio-Rad Laboratories (Hercules, CA, US), or Absea Biotechnology (Bejing, CN). Fibrinogen was procured from CSL Behring (King of Prussia, PA, US) and STA QUALI-CLOT I from Stago (Asnieres, FR). The properties of the tested pads are listed in Table 1.

### Whole Blood Sample Preparation

Whole blood samples were sourced from the Austrian Red Cross in EDTA tubes, which was approved by its ethical review committee. To obtain plasma, blood was centrifuged at 2500 RCF for 15 min at room temperature. After that, the plasma fraction was collected, and a second centrifugation step was carried out by applying 2500 RCF for 10 min to collect the remaining plasma. The left erythrocytes were split into Eppendorf tubes and centrifuged at 100 RCF for 15 min. Blood samples with different hematocrit values were prepared by combining varying ratios of erythrocytes to pure plasma. In addition, the hematocrit values were characterized by an OX-360 cell counter (Balio Diagnostics, Bidart, FR) provided by the Ludwig Boltzmann Institute for Traumatology.

### Detection Pad Characterization

Three nitrocellulose membranes (Immunopore FP, Immunopore RP, and AE99) were prepared with three widths (5 × 30, 3 × 50, and 2 × 75 mm). The membrane strips were immobilized on adhesive tape (ARflow 90469). For determining the flow profile, 10 μL of buffer (HBSS) or plasma was dropped on the membrane, and a video was recorded for later data analysis using open-source image processing software FIJI.

For the blocking buffer, bovine serum albumin (BSA, 0.1 and 1% w/v) was dissolved in PBS or distilled water. Membrane strips of immunopore RP with a width of 3 mm were immersed in the blocking solution for 15 min. After that, the strips were washed twice in PBS or distilled water and dried overnight. Then, the strips were immobilized on ARflow 90469 before adding 10 μL HBSS. A video was recorded and analyzed using open-source image processing software FIJI.

### Conjugation of AuNPs with Antibodies

For the conjugation of 200 μL AuNPs (OD1), 8 μL 0.1 M borate buffer with pH 9 or PBS with pH 7.4 was added. Subsequently, 4 μL of human fibrinogen monoclonal antibody was added with a concentration of 0.1, 0.5, or 1 mg/mL. This mix was incubated on a ThermoMixer C (Eppendorf, Hamburg, DE) for 30 min at 700 rpm, followed by 15 min centrifugation (1400 RCF). The supernatant was discarded, and AuNPs were resuspended in 0.1% BSA w/v in PBS. A sample of AuNPs was mixed with an equal amount of 10% w/v sodium chloride (NaCl), and absorbance was measured (spectrum scan with a step width of 5 nm).

### Conjugate Pad Characterization

Three different conjugate pads (ST14, ST17, Fusion 5) were prepared with a biopsy puncher (diameter of 3 mm). Commercially available AuNPs conjugated with streptavidin were used for the release studies. The conjugates were centrifuged (1400 RCF, 10 min) and resuspended in PBST (PBS with 0.05% TWEEN® 20) or borate buffer (pH 9, 5 mM) with 10% sucrose, respectively, to obtain an optical density of 5 (OD5). Then, each pad was wicked with 3 μL of the OD5 conjugate solution and dried overnight at room temperature. The next day, a test strip was assembled and release was initiated with 10 μL of HBSS. Images were taken before and after releasing the conjugates (as soon as the pads were dried). The intensity of the pads was analyzed with the open-source image processing software FIJI.

### Sample Pad Characterization and PoC Hematocrit Test

Four blood separation pads (GF, GX, GR—Vivid^TM^ plasma separation membrane from Pall; LF1—Whatman^TM^ blood separator from Cytiva) with a size of 3 × 55 mm were prepared using a paper cutter (Novus Dahle, Lingen, DE) and the strips immobilized on the adhesive tape ARflow 90469. For the blood uptake time, 20 μL of blood was added with a pipet and the timer started after the pipet was emptied. As soon as no excess blood was visible, the timer was stopped. To determine the plasma generation capability of the different pads, 20 μL of blood was applied to the strip, and images were taken as soon as the flow stopped. For the PoC hematocrit test, LF1 strips with a size of 3 × 30 mm were placed on the adhesive ARflow 90469. To test the samples with different hematocrit values, 10 μL of each sample was applied to the LF1 membrane using heparinized capillaries (Servoprax, Wesel, DE). The lengths of the red blood cell area and plasma were analyzed with the open-source image processing software FIJI.

### Enzyme-Linked Immunosorbent Assay

A high-binding microplate (Greiner Bio-One, Kremsmünster, AT) was coated with 2 μg/mL of capture antibody overnight and then blocked with 1% BSA. A dilution series of fibrinogen was incubated, followed by a 4 μg/mL biotinylated detection antibody. Then, streptavidin–HRP was incubated to analyze the presence of fibrinogen enzymatically. HRP converted 3,3′,5,5′-tetramethylbenzidine, and after stopping the reaction, the absorbance was measured with the plate reader EnSpire 2300 (PerkinElmer, Waltham, MA, US). Between all incubation steps, the wells were washed multiple times.

### LFA Preparation and Testing

Fibrinogen capture antibodies were dispensed on the nitrocellulose membrane using the AD1520^TM^ Aspirate Dispense System (BioDot, Irvine, CA, US). After that, the membrane was dried at 37 °C for 1 h, then blocked in 1% BSA for 15 min, and washed twice in PBST. Finally, the membrane was dried for 1 h at 37 °C and stored in the fridge until further use. AuNPs with streptavidin were modified with 100 μg/mL biotinylated detection antibodies before adding conjugate buffer (PBST) and drying the conjugates in the conjugate pad. For the LFA assembly, nitrocellulose strips with a width of 2.5 mm were prepared and placed on an adhesive. On one end, a conjugate pad with stored AuNP conjugates and the sample pad was added, and at the other end, the absorbance pad was attached. For testing, different fibrinogen dilutions (2.5, 2, 1.5, 1, 0.5, and 0.25 mg/mL) were prepared in PBS, and reference plasma (STA QUALI-CLOT I) with a known fibrinogen concentration of 2.475 mg/mL was diluted with PBS. For the LFA, 10 μL of the sample (fibrinogen dilution or reference plasma) was added to an Eppendorf tube containing 20 μL of PBS and, after that, applied to the LFA strip. For quantitative analysis, the LFA strips were imaged using a Molecular Imager ChemiDoc XRS System (Bio-Rad Laboratories, Hercules, CA, US) with the Image Lab Software. The line intensity was analyzed with the open-source image processing software FIJI.

### Statistical Analysis

Statistical data analysis and graph preparations were performed with GraphPad Prism 9. To identify single outliers, a Grubb’s test was performed. Statistical significance was determined by performing a Welch’s *t-*test, and normality was assessed by the combination of a Shapiro–Wilk test and Kolmogorov–Smirnov test. Significances were classified as follows: 0.12 (ns.), 0.033 (*), 0.002 (**), < 0.001 (***).

## Supporting Information Available

Additional content regarding the proposed workflow; blood uptake time of different sample pads; experimental setup of detection pad characterization; gold nanoparticle storage in the conjugate pad; and experimental setup and casing of the PoC hematocrit test (PDF)

## References

[ref1] Kasetsirikul S.; Shiddiky M. J. A.; Nguyen N. T. Challenges and Perspectives in the Development of Paper-Based Lateral Flow Assays. Microfluid. Nanofluid. 2020, 24 (2), 1–18. 10.1007/s10404-020-2321-z.

[ref2] Wu G.; Muhammad; Zaman H. Low-Cost Tools for Diagnosing and Monitoring HIV Infection in Low-Resource Settings. Bull. World Health Organ. 2012, 90, 914–920. 10.2471/BLT.12.102780. 23284197 PMC3524957

[ref3] Singer J. M.; Plotz C. M. The Latex Fixation Test: I. Application to the Serologic Diagnosis of Rheumatoid Arthritis. Am. J. Med. 1956, 21 (6), 888–892. 10.1016/0002-9343(56)90103-6. 13372565

[ref4] Yalow R. S.; Berson S. A. Immunoassay of Endogenous Plasma Insulin in Man. J. Clin. Invest. 1960, 39 (7), 1157–1175. 10.1172/JCI104130. 13846364 PMC441860

[ref5] Yetisen A. K.; Akram M. S.; Lowe C. R. Paper-Based Microfluidic Point-of-Care Diagnostic Devices. Lab Chip 2013, 13, 2210 10.1039/c3lc50169h. 23652632

[ref6] Di Nardo F.; Chiarello M.; Cavalera S.; Baggiani C.; Anfossi L. Ten Years of Lateral Flow Immunoassay Technique Applications: Trends, Challenges and Future Perspectives. Sensors 2021, 21 (15), 5185 10.3390/s21155185. 34372422 PMC8348896

[ref7] Zhang L.; Du X.; Su Y.; Niu S.; Li Y.; Liang X.; Luo H. Quantitative Assessment of AD Markers Using Naked Eyes: Point-of-Care Testing with Paper-Based Lateral Flow Immunoassay. J. Nanobiotechnol. 2021, 19 (1), 1–17. 10.1186/S12951-021-01111-Z/TABLES/2. PMC8597216 34789291

[ref8] Huang L.; Tian S.; Zhao W.; Liu K.; Ma X.; Guo J. Multiplexed Detection of Biomarkers in Lateral-Flow Immunoassays. Analyst 2020, 145 (8), 2828–2840. 10.1039/C9AN02485A. 32219225

[ref9] Sohrabi H.; Majidi M. R.; Fakhraei M.; Jahanban-Esfahlan A.; Hejazi M.; Oroojalian F.; Baradaran B.; Tohidast M.; Guardia M. de la; Mokhtarzadeh A. Lateral Flow Assays (LFA) for Detection of Pathogenic Bacteria: A Small Point-of-Care Platform for Diagnosis of Human Infectious Diseases. Talanta 2022, 243, 123330 10.1016/j.talanta.2022.123330. 35272153

[ref10] Sadeghi P.; Sohrabi H.; Hejazi M.; Jahanban-Esfahlan A.; Baradaran B.; Tohidast M.; Majidi M. R.; Mokhtarzadeh A.; Tavangar S. M.; de la Guardia M. Lateral Flow Assays (LFA) as an Alternative Medical Diagnosis Method for Detection of Virus Species: The Intertwine of Nanotechnology with Sensing Strategies. TrAC, Trends Anal. Chem. 2021, 145, 116460 10.1016/j.trac.2021.116460. PMC8529554 34697511

[ref11] Xing K. Y.; Shan S.; Liu D. F.; Lai W. H. Recent Advances of Lateral Flow Immunoassay for Mycotoxins Detection.. TrAC, Trends Anal. Chem. 2020, 133, 116087 10.1016/j.trac.2020.116087.

[ref12] Ngom B.; Guo Y.; Wang X.; Bi D. Development and Application of Lateral Flow Test Strip Technology for Detection of Infectious Agents and Chemical Contaminants: A Review. Anal. Bioanal. Chem. 2010, 397, 1113–1135. 10.1007/s00216-010-3661-4. 20422164

[ref13] Zhou Y.; Wu Y.; Ding L.; Huang X.; Xiong Y. Point-of-Care COVID-19 Diagnostics Powered by Lateral Flow Assay. TrAC, Trends Anal. Chem. 2021, 145, 116452 10.1016/j.trac.2021.116452. PMC8487324 34629572

[ref14] Posthuma-Trumpie G. A.; Korf J.; Van Amerongen A. Lateral Flow (Immuno)Assay: Its Strengths, Weaknesses, Opportunities and Threats. A Literature Survey. Anal. Bioanal. Chem. 2008, 393, 569–582. 10.1007/s00216-008-2287-2. 18696055

[ref15] Shirshahi V.; Liu G. Enhancing the Analytical Performance of Paper Lateral Flow Assays: From Chemistry to Engineering. TrAC, Trends Anal. Chem. 2021, 136, 116200 10.1016/j.trac.2021.116200.

[ref16] Nguyen V. T.; Song S.; Park S.; Joo C. Recent Advances in High-Sensitivity Detection Methods for Paper-Based Lateral-Flow Assay. Biosens. Bioelectron. 2020, 152, 112015 10.1016/j.bios.2020.112015. 32056735

[ref17] Bishop J. D.; Hsieh H. V.; Gasperino D. J.; Weigl B. H. Sensitivity Enhancement in Lateral Flow Assays: A Systems Perspective. Lab Chip 2019, 19, 2486 10.1039/c9lc00104b. 31251312

[ref18] Sadeghi P.; Sohrabi H.; Hejazi M.; Jahanban-Esfahlan A.; Baradaran B.; Tohidast M.; Majidi M. R.; Mokhtarzadeh A.; Tavangar S. M.; de la Guardia M. Lateral Flow Assays (LFA) as an Alternative Medical Diagnosis Method for Detection of Virus Species: The Intertwine of Nanotechnology with Sensing Strategies. TrAC, Trends Anal. Chem. 2021, 145, 116460 10.1016/j.trac.2021.116460. PMC8529554 34697511

[ref19] Deng Y.; Jiang H.; Li X.; Lv X. Recent Advances in Sensitivity Enhancement for Lateral Flow Assay. Microchim. Acta 2021, 188 (11), 1–15. 10.1007/S00604-021-05037-Z/TABLES/1. PMC8513549 34647157

[ref20] Hsieh H.; Dantzler J.; Weigl B. Analytical Tools to Improve Optimization Procedures for Lateral Flow Assays. Diagnostics (Basel) 2017, 7 (2), 29 10.3390/diagnostics7020029. 28555034 PMC5489949

[ref21] Parolo C.; Sena-Torralba A.; Bergua J. F.; Calucho E.; Fuentes-Chust C.; Hu L.; Rivas L.; Álvarez-Diduk R.; Nguyen E. P.; Cinti S.; Quesada-González D.; Merkoçi A. Tutorial: Design and Fabrication of Nanoparticle-Based Lateral-Flow Immunoassays. Nat. Protoc. 2020, 15, 3788–3816. 10.1038/s41596-020-0357-x. 33097926

[ref22] Bialkower M.; McLiesh H.; Manderson C. A.; Tabor R. F.; Garnier G. Rapid Paper Diagnostic for Plasma Fibrinogen Concentration. Analyst 2019, 144 (16), 4848–4857. 10.1039/C9AN00616H. 31294736

[ref23] Rourke C.; Curry N.; Khan S.; Taylor R.; Raza I.; Davenport R.; Stanworth S.; Brohi K. Fibrinogen Levels during Trauma Hemorrhage, Response to Replacement Therapy, and Association with Patient Outcomes. J. Thromb. Haemostasis. 2012, 10 (7), 1342–1351. 10.1111/j.1538-7836.2012.04752.x. 22519961

[ref24] Hofer S.; Schlimp C. J.; Casu S.; Grouzi E. Management of Coagulopathy in Bleeding Patients. J. Clin. Med. 2022, 11, 1 10.3390/jcm11010001. PMC8745606 35011742

[ref25] So̷rensen B.; Tang M.; Larsen O. H.; Laursen P. N.; Fenger-Eriksen C.; Rea C. J. The Role of Fibrinogen: A New Paradigm in the Treatment of Coagulopathic Bleeding. Thromb. Res. 2011, 128 (Suppl. 1), S13–S16. 10.1016/S0049-3848(12)70004-X. 22221845

[ref26] Guven B.; Can M.; Tekin A. Comparison of Fibrinogen Concentrations Determined by the Clauss Method with Prothrombin-Derived Measurements on an Automated Coagulometer. J. Appl. Lab. Med. 2022, 7 (6), 1337–1345. 10.1093/jalm/jfac066. 35993826 PMC9452101

[ref27] Curry N. S.; Davenport R.; Pavord S.; Mallett S. V.; Kitchen D.; Klein A. A.; Maybury H.; Collins P. W.; Laffan M. The Use of Viscoelastic Haemostatic Assays in the Management of Major Bleeding: A British Society for Haematology Guideline. Br. J. Hamaetol. 2018, 182, 789–806. 10.1111/bjh.15524. 30073664

[ref28] Guan Y.; Zhang K.; Xu F.; Guo R.; Fang A.; Sun B.; Meng X.; Liu Y.; Bai M. An Integrated Platform for Fibrinogen Quantification on a Microfluidic Paper-Based Analytical Device. Lab Chip 2020, 20 (15), 2724–2734. 10.1039/D0LC00439A. 32588856

[ref29] Saidykhan J.; Selevic L.; Cinti S.; May J. E.; Killard A. J. Paper-Based Lateral Flow Device for the Sustainable Measurement of Human Plasma Fibrinogen in Low-Resource Settings. Anal. Chem. 2021, 93 (41), 14007–14013. 10.1021/acs.analchem.1c03665. 34615344 PMC8529579

[ref30] Bialkower M.; Manderson C. A.; McLiesh H.; Tabor R. F.; Garnier G. Paper Diagnostic for Direct Measurement of Fibrinogen Concentration in Whole Blood. ACS Sens. 2020, 5 (11), 3627–3638. 10.1021/ACSSENSORS.0C01937/ASSET/IMAGES/MEDIUM/SE0C01937_M001.GIF. 33095567

[ref31] Bialkower M.; McLiesh H.; Manderson C. A.; Tabor R. F.; Garnier G. Rapid, Hand-Held Paper Diagnostic for Measuring Fibrinogen Concentration in Blood. Anal. Chim. Acta 2020, 1102, 72–83. 10.1016/j.aca.2019.12.046. 32043998

[ref32] Li H.; Han D.; Pauletti G. M.; Hegener M. A.; Steckl A. J. Correcting the Effect of Hematocrit in Whole Blood Coagulation Analysis on Paper-Based Lateral Flow Device. Anal. Methods 2018, 10 (24), 2869–2874. 10.1039/C8AY00192H.

[ref33] Frantz E.; Li H.; Steckl A. J. Quantitative Hematocrit Measurement of Whole Blood in a Point-of-Care Lateral Flow Device Using a Smartphone Flow Tracking App. Biosens. Bioelectron. 2020, 163, 112300 10.1016/j.bios.2020.112300. 32568698

[ref34] Berry S. B.; Fernandes S. C.; Rajaratnam A.; Dechiara N. S.; Mace C. R. Measurement of the Hematocrit Using Paper-Based Microfluidic Devices. Lab Chip 2016, 16 (19), 3689–3694. 10.1039/C6LC00895J. 27604182

[ref35] Fernandes S. C.; Baillargeon K. R.; MacE C. R. Reduction of Blood Volume Required to Perform Paper-Based Hematocrit Assays Guided by Device Design. Anal. Methods 2019, 11 (15), 2057–2063. 10.1039/C9AY00010K.

[ref36] Kim S. C.; Jalal U. M.; Im S. B.; Ko S.; Shim J. S. A Smartphone-Based Optical Platform for Colorimetric Analysis of Microfluidic Device. Sens. Actuators, B 2017, 239, 52–59. 10.1016/j.snb.2016.07.159.

[ref37] Anjali N.; Das S.; Chakraborty S. Simultaneous Quantitative Detection of Hematocrit and Hemoglobin from Whole Blood Using a Multiplexed Paper Sensor with a Smartphone Interface. Lab Chip 2023, 23 (2), 318–329. 10.1039/D2LC00456A. 36562505

[ref38] Liao Y. M.; Chiu P. Y.; Chien Y. S.; Chen C. F. Music Box-Inspired Semi-Automatic Hematocrit Validation Device. ACS Sens. 2023, 8 (8), 2952–2959. 10.1021/acssensors.3c00129. 37418365

[ref39] Rossaint R.; Afshari A.; Bouillon B.; Cerny V.; Cimpoesu D.; Curry N.; Duranteau J.; Filipescu D.; Grottke O.; Gro̷nlykke L.; Harrois A.; Hunt B. J.; Kaserer A.; Komadina R.; Madsen M. H.; Maegele M.; Mora L.; Riddez L.; Romero C. S.; Samama C. M.; Vincent J. L.; Wiberg S.; Spahn D. R. The European Guideline on Management of Major Bleeding and Coagulopathy Following Trauma: Sixth Edition. Crit. Care 2023, 27 (1), 80 10.1186/s13054-023-04327-7. 36859355 PMC9977110

[ref40] Božič D.; Sitar S.; Junkar I.; Štukelj R.; Pajnič M.; Žagar E.; Kralj-Iglič V.; Kogej K. Viscosity of Plasma as a Key Factor in Assessment of Extracellular Vesicles by Light Scattering. Cells 2019, 8 (9), 1046 10.3390/cells8091046. 31500151 PMC6769602

[ref41] Christau S.; Moeller T.; Genzer J.; Koehler R.; Von Klitzing R. Salt-Induced Aggregation of Negatively Charged Gold Nanoparticles Confined in a Polymer Brush Matrix. Macromolecules 2017, 50 (18), 7333–7343. 10.1021/acs.macromol.7b00866.

[ref42] Zhang L.; Mazouzi Y.; Salmain M.; Liedberg B.; Boujday S. Antibody-Gold Nanoparticle Bioconjugates for Biosensors: Synthesis, Characterization and Selected Applications. Biosens. Bioelectron. 2020, 165, 112370 10.1016/j.bios.2020.112370. 32729502

[ref43] Kaur M.; Eltzov E. Optimizing Effective Parameters to Enhance the Sensitivity of Vertical Flow Assay for Detection of Escherichia Coli. Biosensors 2022, 12 (2), 63 10.3390/bios12020063. 35200324 PMC8869093

