# Supplementary material for: Development of a Paper-based Hematocrit Test and a Lateral Flow Assay to Detect Critical Fibrinogen Concentrations Using a Bottom-Up Pyramid Workflow Approach
Source: ACS Omega. 2024 Feb 5;9(7):8533–42. doi: 10.1021/acsomega.3c10045 (PMC10882670; doi:10.1021/acsomega.3c10045)
Supplement: Supplementary file 1 — ao3c10045_si_001.pdf [file ao3c10045_si_001.pdf]

Supplementary Information

Development of a Paper-based Hematocrit Test and a Lateral Flow Assay to Detect Critical Fibrinogen Concentrations Using a Bottom-up Pyramid Workflow Approach

Silvia Schobesberger[1], Helena Thumfart[1], Florian Selinger[1], Christoph J. Schlimp[2,3], Johannes Zipperle[2] and Peter Ertl[1*]

[1] TU Wien, Faculty of Technical Chemistry, Getreidemarkt 9, 1060 Vienna, Austria

[2] Ludwig-Boltzmann-Institute for Traumatology, the research center in cooperation with AUVA, Donaueschingenstraße 13, 1200 Vienna, Austria

[3] Department of Anaesthesiology and Intensive Care, AUVA Trauma Center Linz, Garnisonstraße 7, 4010 Linz, Austria

General description of the LFA development workflow

We propose a pyramid-based approach where the fundament consisting of sample matrix, sample volume, limit of detection and assay time is defined. In general, lateral flow assays (LFAs) usually consist of 4 different pads facilitating distinct functions: (1) sample pad for sample application and preparation, (2) conjugate pad for storage of labeled detection reagents, (3) detection pad with immobilized capture probes for test and control line and (4) absorbance pad for soaking excess liquid.[1–4] Thus, a set of membranes for each pad needs to be investigated for each matrix requirements. As an example, blood needs a membrane capable of separating red blood cells as well as a detection pad that is suitable for plasma. In addition, different membrane widths of the nitrocellulose membrane need to be studied, since thickness and layout effect the flow behavior.[5] For the read-out, detection reagents need to be conjugated with labels or novel labels developed and storage in the conjugate pad investigated. Once the testing in stage 1 is completed, pad arrangement is adjusted in accordance with the assay optimization since, as an example, a competitive assay might require and additional stacking pad to ensure interaction time.[4] After that, the final assembly in stage 3 includes the development of a casing and read-out unit, depending on the aim of the study. It is important to also keep the applicability for the end-user in mind, where blood samples are applied and when the tests are finished, the results may be analyzed using a PoC reader (e.g. smartphone).

Additional data and experimental setup

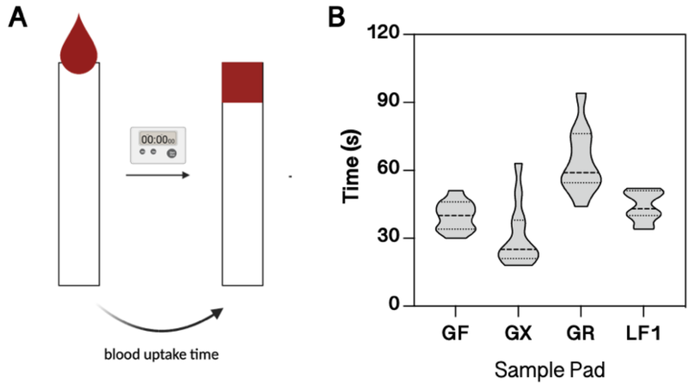

Figure S1: Blood uptake time of different sample pads. A) Schematic illustration of employed method. A drop of blood was added and the time measured until the drop was soaked into the pad. B) Blood uptake time of different sample pads (n = 11).

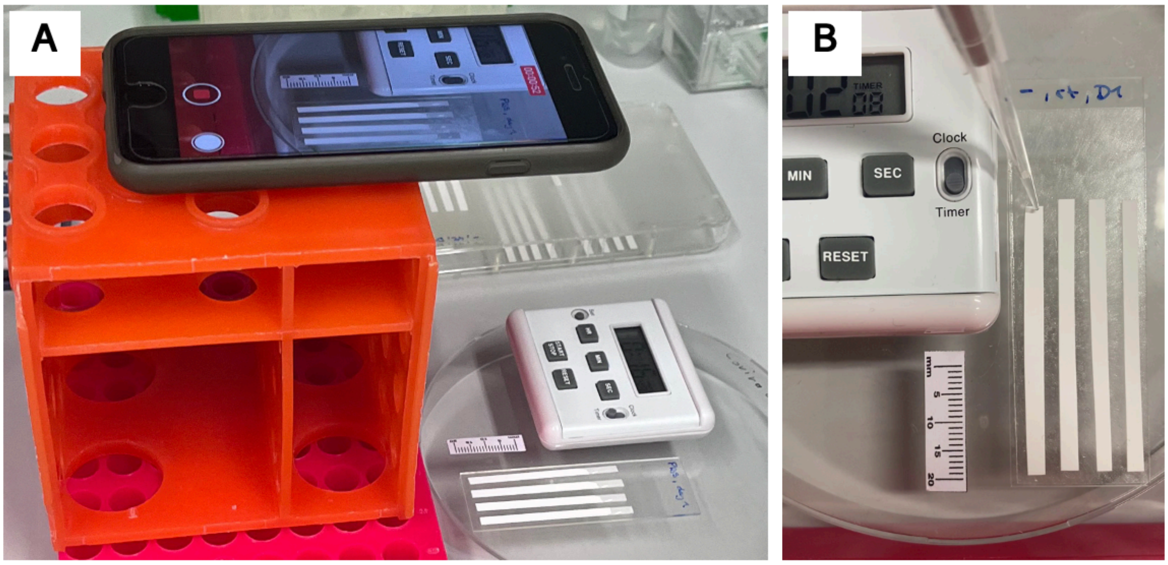

Figure S2: A) Measurement set-up to determine the travel distance of buffer or plasma. B) Exemplary image used for data analysis.

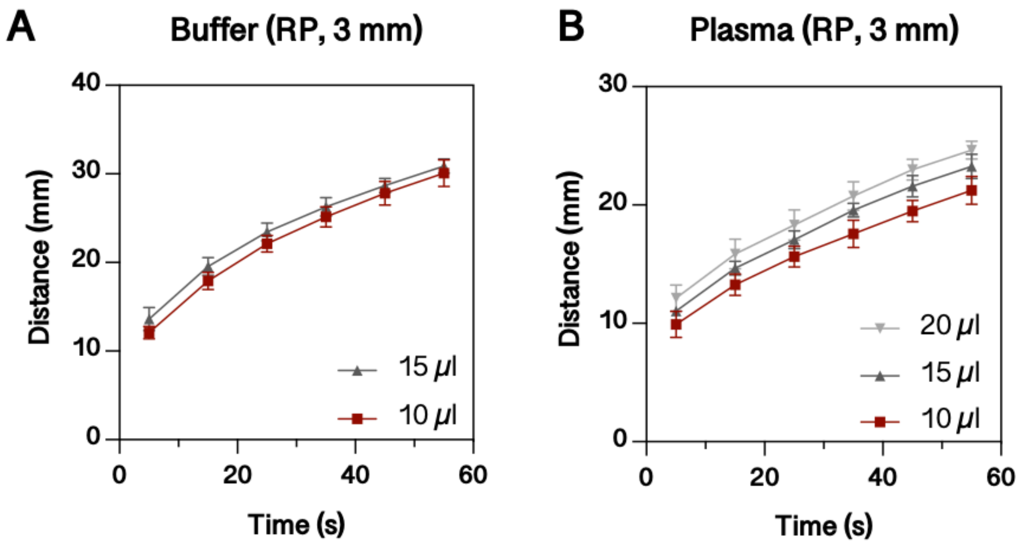

Figure S3: Flow profile of nitrocellulose membrane RP when different volumes of A) buffer or B) plasma are applied.

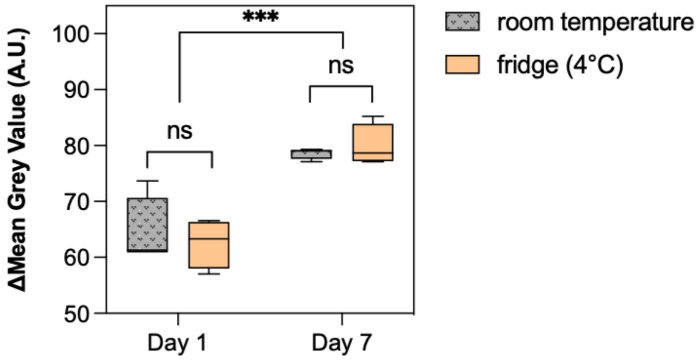

Figure S4: Comparison of gold nanoparticle (AuNP) release after storage in fridge or at room temperature, measured on day 1 and day 7. Statistical significance by two-way ANOVA (n=4).

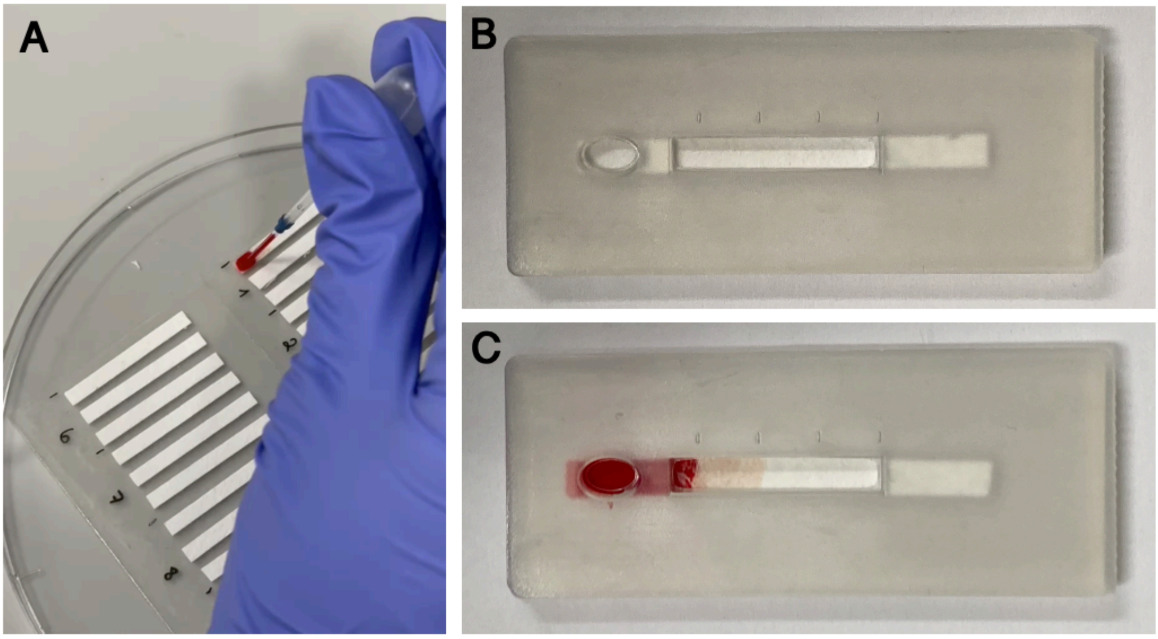

Figure S5: A) Testing of different hematocrits by application of 10 µl blood. B) 3D printed casing for hematocrit test before and C) after blood application.

References

(1) Di Nardo, F.; Chiarello, M.; Cavalera, S.; Baggiani, C.; Anfossi, L. Ten Years of Lateral Flow Immunoassay Technique Applications: Trends, Challenges and Future Perspectives. *Sensors* **2021**, *21* (15), 5185. https://doi.org/10.3390/S21155185.

(2) Shirshahi, V.; Liu, G. Enhancing the Analytical Performance of Paper Lateral Flow Assays: From Chemistry to Engineering. *TrAC, Trends Anal. Chem.* **2021**, *136*, 116200. https://doi.org/10.1016/J.TRAC.2021.116200.

(3) Quesada-González, D.; Merkoçi, A. Nanoparticle-Based Lateral Flow Biosensors. *Biosens. Bioelectron.* **2015**, *73*, 47–63. https://doi.org/10.1016/J.BIOS.2015.05.050.

(4) Tsai, T. T.; Huang, T. H.; Chen, C. A.; Ho, N. Y. J.; Chou, Y. J.; Chen, C. F. Development a Stacking Pad Design for Enhancing the Sensitivity of Lateral Flow Immunoassay. *Sci Rep* **2018**, *8* (1). https://doi.org/10.1038/s41598-018-35694-9.

(5) Fu, E.; Lutz, B.; Kauffman, P.; Yager, P. Controlled Reagent Transport in Disposable 2D Paper Networks. *Lab Chip* **2010**, *10* (7), 918–920. https://doi.org/10.1039/b919614e.
